# Coordinating innovation without unified authority: leadership and organizational identification in hybrid organizations

**DOI:** 10.3389/fpsyg.2026.1803166

**Published:** 2026-04-02

**Authors:** Yao Fu, Yun-Hua Zhong, Agudamu, Wei-Long Zhang

**Affiliations:** 1 College of Educational Sciences, Hunan Normal University, Changsha, China; 2 Xiangtan Institute of Science and Technology, Xiangtan, China; 3 Graduate School of Social Welfare, Sungkyunkwan University, Seoul, Republic of Korea; 4 College of Physical Education, Hunan Normal University, Changsha, China

**Keywords:** apprenticeship systems, boundary spanning, innovation domains, institutional plurality, sensemaking

## Abstract

We examine patterns of coordinated innovation in hybrid organizations characterized by institutional plurality, fragmented authority, and overlapping role expectations by studying an apprenticeship system jointly governed by educational institutions and enterprises. Drawing on social identity theory as well as research on transformational leadership and organizational identification, we propose that leadership is associated with innovation not only through direct relationships but also through shared identification that aligns actors embedded in multiple institutional domains. We further suggest that these relationships vary across innovation domains. Using survey data from apprentices (*N* = 2,156) within Geely, a leading innovation-intensive automotive enterprise, we test a mediation model distinguishing managerial and technological innovation. The findings indicate that organizational identification partially mediates the association between transformational leadership and innovation, with systematic domain differences. Identification exhibits a stronger association with technological innovation, whereas transformational leadership shows a comparatively stronger direct association with managerial innovation. These findings highlight how leadership and identification are linked to coordination processes in hybrid organizations operating under high innovation intensity. By theorizing identity-based mechanisms consistent with social identity theory underlying domain-contingent innovation patterns, this study advances understanding of leadership and innovation in hybrid organizational forms and sheds light on coordination under institutional plurality.

## 1 Introduction

Hybrid organizations that span multiple institutional domains have become a defining organizational form in contemporary economies. Universities collaborate with firms to co-produce knowledge and skills, public agencies partner with private enterprises to deliver social services, and educational institutions increasingly share governance responsibilities with industry actors to address workforce transformation. These arrangements are widely regarded as vehicles for innovation because they combine heterogeneous resources, expertise, and institutional logics (Battilana and Lee, 2014; Besharov and Smith, 2014). Yet empirical evidence shows that hybridity also generates persistent coordination problems. Despite formal agreements, policy support, and substantial investments, many hybrid organizations struggle to translate collaboration into sustained organizational innovation (Vikkelsø et al., 2021). This tension raises a fundamental theoretical puzzle: how do hybrid organizations enable coordinated innovation when authority structures, role expectations, and organizational identities are fragmented across institutional boundaries?

Research on hybrid organizations has primarily approached this puzzle from a structural and institutional perspective. Prior studies emphasize governance arrangements, selective coupling, and the management of competing institutional logics as central determinants of hybrid performance (Battilana and Dorado, 2010; Pache and Santos, 2013; Reuer and Devarakonda, 2016). While these accounts illuminate how hybridity is designed and stabilized at the macro level, they devote comparatively less attention to the micro-level processes through which coordination and innovation are enacted in everyday organizational life. In particular, leadership is often treated as contextual or exogenous rather than as a relational mechanism through which institutional plurality is rendered workable. As a result, we know relatively little about how leaders enable coordinated innovation when authority is distributed and identities are overlapping rather than singular.

Leadership research—especially the extensive literature on transformational leadership—offers partial but incomplete insight into this question. Transformational leadership has been consistently associated with creativity, learning, and innovation (Gumusluoğlu and Ilsev, 2009; Jiang and Chen, 2018). By articulating an inspiring vision and fostering trust, transformational leaders mobilize followers toward change-oriented goals (Podsakoff et al., 1990; Walumbwa et al., 2008). Organizational identification has been theorized as a central mechanism in this process, enhancing alignment and discretionary effort (Ashforth and Mael, 1989; Riketta, 2005).

However, the dominant paradigm of “transformational leadership → identification → innovation” implicitly presupposes organizational contexts characterized by relatively unified authority structures and coherent identity frameworks. Within such settings, identification strengthens alignment with already established goals, and leadership operates largely through motivational amplification. Hybrid organizations systematically challenge these assumptions. Authority is distributed across institutional domains, evaluative criteria are pluralistic, and actors navigate multiple, partially conflicting identities. Under such conditions, alignment cannot be assumed—it must be constructed.

This study therefore moves beyond applying an established model to a new context. Instead, we test and revise its boundary conditions. We argue that in hybrid organizations, transformational leadership operates primarily as a process of sensemaking and identity integration. Rather than merely inspiring followers within a stable system, leaders construct shared meaning that reduces ambiguity, reconciles competing institutional logics, and stabilizes expectations across fragmented roles. Organizational identification, from this perspective, functions not simply as an attitudinal attachment but as a coordination infrastructure that enables collective innovation under institutional plurality.

To examine this argument, we studied a large-scale apprenticeship system jointly governed by educational institutions and enterprises. Apprenticeship arrangements embody core features of hybridity: shared authority, role asymmetry, and sustained interaction among actors embedded in distinct institutional logics. The Chinese apprenticeship system offers a theoretically revealing setting because rapid institutionalization coexists with substantial variation in organizational effectiveness. This context allows us to investigate how leadership and identity processes enable coordinated innovation when authority is distributed and identities are plural.

By reconceptualizing transformational leadership as an identity-integrating and sensemaking mechanism in hybrid organizations, this study advances leadership theory, hybrid organization research, and innovation scholarship. It demonstrates that coordinated innovation under institutional plurality depends not only on structural design but also on leaders’ capacity to construct shared meaning across institutional boundaries.

## 2 Literature review

### 2.1 Theoretical foundation: social identity processes in hybrid organizations

The theoretical foundation of this study draws on social identity theory, which explains how individuals define themselves through their membership in social groups and organizations (Ashforth and Mael, 1989). According to social identity theory, organizational identification emerges when individuals perceive a sense of oneness with an organization and incorporate organizational membership into their self-concept. This identification shapes attitudes and behaviors by aligning individual motivations with collective goals, thereby facilitating cooperation, coordination, and commitment to shared objectives.

Leadership plays a central role in shaping identity processes within organizations. Research on identity-based leadership suggests that leaders influence followers not only through formal authority or incentives but also by constructing shared meanings about “who we are” as a collective and what the organization stands for Kark et al. (2003). By articulating a compelling vision and emphasizing collective values, leaders can strengthen organizational identification and mobilize members toward coordinated action.

These identity processes become particularly important in hybrid organizations, where actors operate across multiple institutional domains characterized by distinct norms, values, and authority structures (Reissner, 2019; Cornelissen et al., 2021). Institutional plurality can fragment identity and create ambiguity regarding organizational goals and priorities. In such contexts, coordination cannot rely solely on hierarchical authority or structural arrangements; instead, shared identity and meaning become critical mechanisms through which actors align their behaviors.

Building on this perspective, this study conceptualizes transformational leadership as a mechanism that facilitates identity integration and collective sensemaking in hybrid organizations. By fostering organizational identification among members embedded in diverse institutional contexts, transformational leadership may enable coordinated innovation despite fragmented authority and pluralistic institutional demands. This theoretical lens provides the foundation for examining how leadership and identification processes jointly influence organizational innovation in hybrid settings.

### 2.2 Hybrid organizations and the challenge of coordinated innovation

Hybrid organizations combine elements from multiple institutional domains and are governed by heterogeneous logics, authority structures, and evaluative criteria (Battilana and Lee, 2014; Besharov and Smith, 2014). Institutional plurality introduces ambiguity regarding priorities and performance standards, which can undermine learning and experimentation (Greenwood et al., 2011; Smith and Besharov, 2019). Unlike unitary organizations, hybrid organizations cannot rely solely on hierarchical authority or shared identity to ensure coordination.

Structural and governance-based mechanisms mitigate overt conflict (Battilana and Dorado, 2010; Pache and Santos, 2013), but they do not fully resolve the interpretive challenges of collaboration. Innovation requires sustained coordination under uncertainty (Anderson et al., 2014). Thus, hybrid organizations face not only structural but interpretive coordination problems.

### 2.3 Apprenticeship-based hybrid organizations as a setting of institutional plurality

Apprenticeship-based hybrid organizations constitute a distinctive and theoretically informative setting in which the coordination challenges associated with institutional plurality are particularly salient. These organizations span educational and industrial domains by integrating training, skill development, and productive work within a single organizational arrangement. As a result, they operate at the intersection of educational, professional, and market logics, each of which carries distinct expectations regarding authority, performance, and legitimacy.

In apprenticeship systems embedded within industry–education collaborations, formal authority structures are often fragmented. Supervisors and mentors may shape apprentices’ learning and daily work practices without possessing full hierarchical control over evaluation, promotion, or long-term career outcomes. At the same time, apprentices and employees occupy multiple social roles, functioning simultaneously as learners, workers, and organizational members. These overlapping roles expose individuals to competing normative expectations and evaluative criteria, complicating how they interpret organizational goals and their position within the organization.

Such institutional complexity has important implications for coordinated innovation. Innovation in apprenticeship-based hybrid organizations typically requires sustained collaboration across occupational, educational, and organizational boundaries. However, the coexistence of multiple identities and institutional logics may weaken shared understanding and attenuate collective commitment to innovation initiatives. Without clear integrative interpretive frames, organizational members may prioritize role-specific or institution-specific objectives, thereby constraining coordinated action.

Under these conditions, leadership may assume a role that extends beyond formal coordination. Leaders in apprenticeship-based hybrid organizations often function simultaneously as managers, mentors, and symbolic representatives of organizational values, engaging in frequent and relationally embedded interactions with members. Through these interactions, leaders may shape how individuals interpret organizational membership, reconcile multiple role identities, and make sense of competing institutional demands. Consequently, leadership processes emphasizing meaning construction, value integration, and relational alignment are likely to be especially salient in this setting.

Apprenticeship-based hybrid organizations therefore provide a revealing context for examining how transformational leadership and organizational identification are associated with coordination under institutional plurality. By foregrounding identity integration and shared purpose, transformational leadership may contribute to aligning members’ efforts toward collective innovation goals despite fragmented authority and pluralistic institutional demands.

### 2.4 Transformational leadership as sensemaking and boundary integration

Transformational leadership has traditionally been conceptualized as a motivational and vision-driven leadership style that enhances followers’ commitment and discretionary effort (Avolio and Bass, 1995). Empirical evidence links transformational leadership to creativity and innovation by highlighting its role in intellectual stimulation, individualized consideration, and inspirational motivation (Gumusluoğlu and Ilsev, 2009; Jiang and Chen, 2018). These effects are typically attributed to leaders’ ability to provide vision, intellectual stimulation, and individualized consideration, which together create conditions conducive to exploration and experimentation.

However, this theoretical model has largely been developed in organizational contexts characterized by relatively coherent authority structures and comparatively unified identity frameworks. In such settings, alignment around collective goals is facilitated by shared evaluative criteria and centralized authority, and transformational leadership operates primarily by amplifying motivation and strengthening commitment within an already integrated system. Organizational identification, in turn, enhances alignment with goals that are institutionally stable and broadly uncontested.

Hybrid organizations systematically challenge these underlying assumptions. Authority is distributed across institutional domains, role expectations are ambiguous, and actors navigate overlapping professional and organizational identities. Under such conditions, collective alignment cannot be taken for granted. Instead, it must be actively constructed through processes that reduce ambiguity and reconcile competing institutional logics.

In hybrid settings, transformational leadership therefore assumes a more foundational function. It becomes a process of sensemaking and identity integration through which leaders construct shared interpretations of purpose, legitimacy, and collective membership. Rather than merely motivating followers within a coherent system, transformational leaders in hybrid organizations must integrate disparate institutional logics into an overarching narrative that renders collaboration meaningful and legitimate. Through articulating a superordinate vision that transcends institutional boundaries, leaders reduce perceived incompatibilities among competing logics and foster cross-boundary alignment (Waldman et al., 2001). Through ongoing relational interactions, they provide interpretive clarity that enables actors to reconcile fragmented roles and commit to collective innovation.

Accordingly, transformational leadership is expected to be positively related to organizational innovation in hybrid organizations.

*Hypothesis 1:* Transformational leadership is positively related to organizational innovation in hybrid organizations.

### 2.5 Organizational identification under conditions of institutional plurality

Organizational identification refers to the degree to which individuals define themselves in terms of their membership in an organization and experience a sense of oneness with it (Ashforth and Mael, 1989). Extensive research has shown that identification enhances cooperation, persistence, and willingness to engage in discretionary behaviors that support organizational goals (Riketta, 2005; Lee et al., 2015). In innovation contexts, organizational identification has been associated with greater knowledge sharing, creative effort, and commitment to collective problem solving (Carmeli et al., 2013).

Yet most of this research assumes a relatively coherent organizational identity. In such contexts, identification strengthens alignment with organizational goals that are institutionally stable and hierarchically reinforced. In hybrid organizations, however, identification processes are more complex because individuals often hold multiple, partially conflicting identities tied to different institutional affiliations, such as profession, organization, or sector (Kreiner et al., 2006). Rather than a single focal object of identification, actors in hybrid settings must navigate identity ambiguity and negotiate which values and goals should guide their behavior.

Under conditions of institutional plurality, organizational identification therefore performs a qualitatively different role. Beyond fostering attachment, identification functions as a coordination mechanism that stabilizes expectations and legitimizes cross-boundary collaboration. By providing a shared interpretive and normative framework, identification reduces uncertainty and enables actors embedded in different institutional domains to align their actions despite fragmented authority structures (Hogg and Terry, 2000; He and Brown, 2013). In this sense, identification serves not merely as an attitudinal outcome but as a relational infrastructure that makes sustained collective innovation possible in hybrid organizations.

### 2.6 Transformational leadership and organizational identification in hybrid organizations

Leadership plays an important role in shaping organizational identification, particularly in contexts characterized by ambiguity and change. Transformational leaders articulate values, model desired behaviors, and emphasize collective goals that transcend individual or subgroup interests (Walumbwa et al., 2008; Kark et al., 2003). Through these processes, leaders may contribute to followers’ construction of a shared understanding of “who we are” and “what we are trying to achieve.”

In hybrid organizations, this identity-shaping function is especially salient. Because institutional plurality can fragment identity and generate competing frames of reference, actors may experience difficulty developing a coherent sense of belonging. Transformational leaders can mitigate this challenge by integrating diverse institutional values into an overarching narrative that legitimizes collaboration and innovation. By emphasizing common purpose and shared outcomes, leaders may reduce identity tension and foster a sense of collective membership that supports coordinated action across boundaries (Hogg et al., 2012).

Accordingly, transformational leadership is expected to strengthen organizational identification in hybrid settings, not merely by enhancing affective attachment, but by providing interpretive clarity and identity integration.

*Hypothesis 2:* Transformational leadership is positively related to organizational identification in hybrid organizations.

### 2.7 Organizational identification and organizational innovation

Organizational identification has long been recognized as a key antecedent of behaviors that support collective outcomes. Identified members tend to demonstrate greater willingness to share knowledge, invest effort in collective problem solving, and persist under uncertainty—behaviors that are frequently associated with organizational innovation (Carmeli et al., 2013; Van Knippenberg, 2000). Identification is also associated with higher levels of trust and psychological safety, which may lower the perceived risks of proposing novel ideas and experimenting with new approaches (Edmondson, 1999).

In hybrid organizations, where innovation often requires collaboration across institutional and professional boundaries, these associations are particularly relevant. Strong organizational identification may reduce intergroup distinctions and facilitate the integration of diverse perspectives, thereby supporting innovation across organizational domains. When members perceive themselves as part of a shared collective, they may be more inclined to coordinate their actions and align their efforts toward joint innovation goals despite differences in institutional affiliation. Therefore, organizational identification is expected to be positively associated with organizational innovation in hybrid organizations.

*Hypothesis 3:* Organizational identification is positively related to organizational innovation in hybrid organizations.

### 2.8 Organizational identification as a mediating mechanism

Integrating the above arguments suggests that organizational identification may function as a central mechanism linking transformational leadership and innovation in hybrid organizations. While transformational leadership may also exhibit direct associations with innovation by encouraging exploration, legitimizing change initiatives, and focusing organizational attention on development-oriented goals, the complexity of hybrid settings may limit the effectiveness of directive influence alone. Institutional plurality increases reliance on shared meaning and identity as coordination devices.

By fostering organizational identification, transformational leadership may contribute to the alignment of fragmented roles and collective innovation goals. Identification provides a social and cognitive foundation for sustained collaboration by fostering shared understanding, trust, and commitment across institutional boundaries. In this way, organizational identification is theorized to mediate the relationship between transformational leadership and organizational innovation under conditions of institutional plurality.

*Hypothesis 4:* Organizational identification mediates the relationship between transformational leadership and organizational innovation in hybrid organizations.

### 2.9 Differentiating managerial and technological innovation

Organizational innovation encompasses multiple forms of change that differ in content, processes, and coordination requirements. Prior research distinguishes between managerial innovation—changes in organizational structures, management practices, and administrative processes—and technological innovation—the development or adoption of new products, services, or production technologies (Birkinshaw et al., 2008; Damanpour and Evan, 1984).

Although both forms of innovation require coordination and collective engagement, they may place distinct demands on leadership and identity-based processes. Managerial and technological innovations differ in their sources of uncertainty, implementation dynamics, and relative reliance on formal authority vs. collective alignment. Accordingly, the associations linking transformational leadership and organizational identification to these innovation domains may vary in magnitude.

Rather than assuming uniform relationships across innovation domains, it is therefore theoretically important to examine whether organizational identification mediates the association between transformational leadership and both managerial and technological innovation, and whether the strength of this mediation differs across domains.

*Hypothesis 5a:* Organizational identification mediates the relationship between transformational leadership and managerial innovation in hybrid organizations.

*Hypothesis 5b:* Organizational identification mediates the relationship between transformational leadership and technological innovation in hybrid organizations.

Although mediation is anticipated for both domains, managerial and technological innovation differ in coordination demands. Managerial innovation often involves adjustments to routines and authority structures, whereas technological innovation frequently entails experimentation, knowledge integration, and cross-boundary collaboration. These differences suggest that the relative importance of identity-based mediation and more direct leadership associations may vary across innovation domains. Rather than advancing a directional prediction, we therefore examine this issue as an open theoretical question:

RQ1. Does the mediating role of organizational identification in the relationship between transformational leadership and innovation differ between managerial and technological innovation?

**Figure 1:**
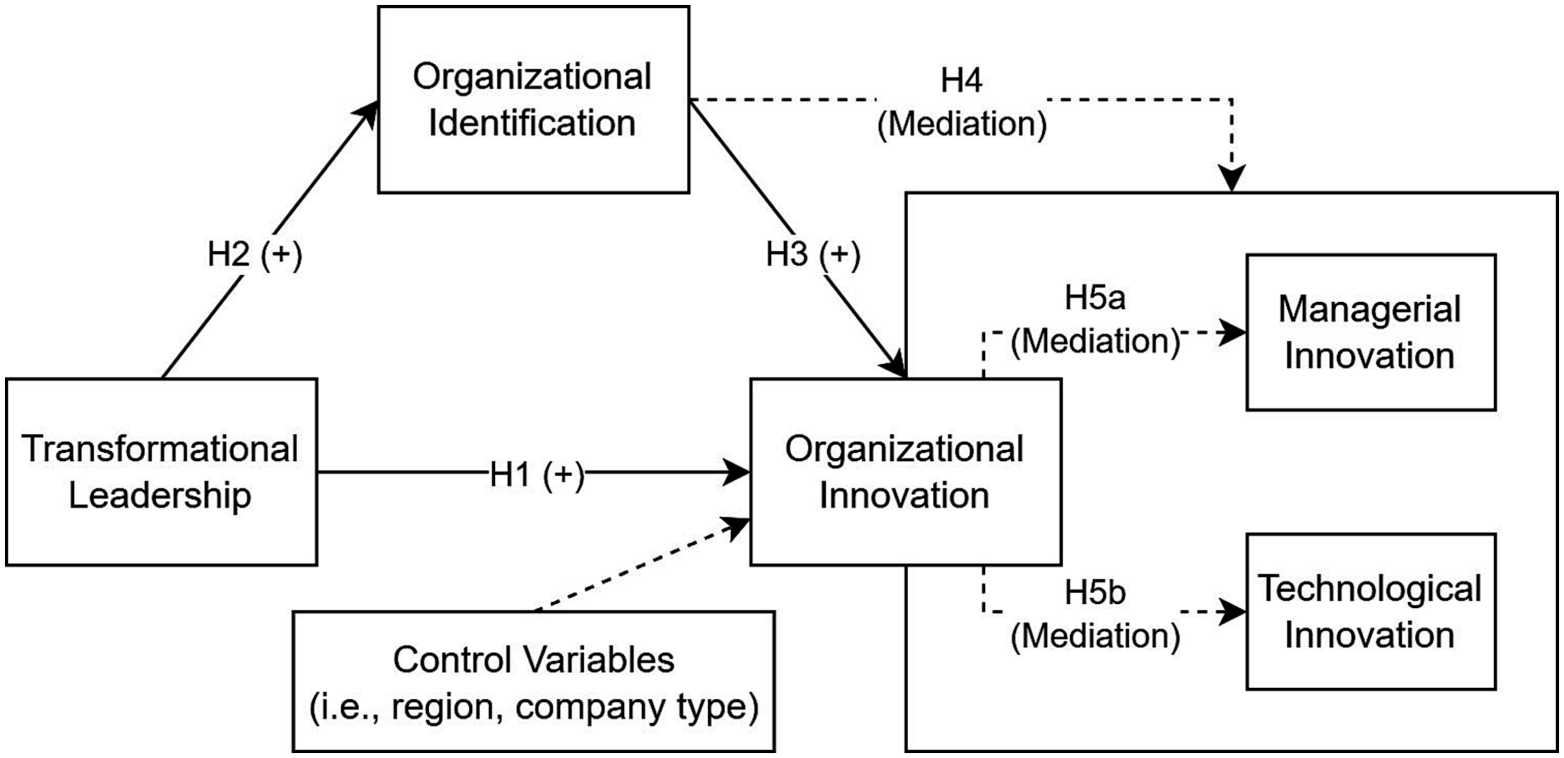
Proposed conceptual model.

Overall, the theoretical relationships proposed in this study are summarized in Figure 1. Drawing on social identity theory, the model conceptualizes transformational leadership as a mechanism through which leaders construct shared meanings and foster organizational identification among members embedded in hybrid organizational settings. Organizational identification, in turn, facilitates coordinated innovation by aligning members’ motivations and behaviors with collective organizational goals. The model therefore proposes that transformational leadership directly influences both managerial and technological innovation, while also exerting indirect effects through the development of organizational identification. In this way, organizational identification functions as a key mediating mechanism linking leadership processes to innovation outcomes within hybrid organizations.

## 3 Methods

### 3.1 Sample and data collection

This study was conducted within Zhejiang Geely Holding Group Co., Ltd. (hereafter, Geely), a leading automobile manufacturer and one of China’s earliest national-level pilot enterprises for industry–education integration. Geely has established extensive apprenticeship-based collaboration networks with more than 300 vocational colleges and applied universities across China, implementing multiple forms of school–enterprise cooperation, including apprenticeship programs, on-site training schools within enterprises, enterprise-based training units within schools, and industry colleges. These arrangements have produced a relatively mature ecosystem of industry–education integration, making Geely’s apprenticeship system a theoretically informative setting for examining coordination, leadership, and innovation in hybrid organizations.

Geely’s apprenticeship programs operate at substantial scale and organizational complexity, encompassing diverse regional contexts, occupational roles, and organizational units. Apprentices are simultaneously embedded in educational institutions and enterprise operations, participating in structured training while engaging in enterprise-based work practices. This dual embeddedness creates conditions of institutional plurality and fragmented authority, rendering leadership and identification processes particularly salient for coordinated innovation.

*A priori* sample-size considerations were guided by model complexity rather than by simple item-count heuristics. Prior methodological research indicates that structural equation models (SEM) typically require approximately 200 cases for relatively simple models, with substantially larger samples recommended for models involving multiple latent constructs, mediation pathways, and bootstrap-based inference (Fritz and MacKinnon, 2007; Wolf et al., 2013). Research on mediation further indicates that statistical power to detect indirect effects is often limited at smaller sample sizes and that bootstrap-based confidence intervals benefit from larger samples (MacKinnon et al., 2004). Accordingly, the study was designed to recruit a sample well above commonly cited lower-bound recommendations to ensure stable parameter estimation and reliable bootstrap-based mediation and cross-domain difference testing.

To ensure the relevance and validity of respondents’ evaluations, participation was restricted to individuals who met three eligibility criteria. Respondents were required to be formally enrolled in Geely’s apprenticeship program, to have entered enterprise work positions or participated in enterprise-based practice for no fewer than two academic semesters, and to be directly involved in day-to-day organizational activities rather than solely classroom-based training. These criteria ensured that respondents had sustained exposure to leadership processes and innovation-related practices within the hybrid organizational context.

To enhance representativeness, the study employed a combination of stratified random sampling and maximum variation sampling. Participants were drawn from 45 collaborating vocational institutions and enterprise subsidiaries, stratified by geographic region and industry segment. This approach ensured coverage of diverse organizational units, functional roles, and regional contexts while avoiding overrepresentation of any single site or occupational category.

Data were collected using the online survey platform Wenjuanxing. The survey link was distributed independently by the researcher (Y. F.). Participation was voluntary, and respondents completed the survey outside formal evaluation or performance review processes. Supervisors and organizational administrators did not have access to individual responses, reducing potential power asymmetries and social desirability concerns.

The survey was administered anonymously, and no personally identifiable information was collected. Respondents were informed of the voluntary nature of participation and the confidentiality of their responses at the beginning of the questionnaire. Because the study involved minimal risk and did not collect sensitive personal data, written informed consent was waived, and consent was implied through voluntary completion of the survey.

### 3.2 Measures

All focal constructs were measured using established multi-item scales drawn from prior research and adapted to the context of apprenticeship-based hybrid organizations. The survey was administered in Chinese, and an English translation of the complete instrument is provided in Supplementary Box 1.

Transformational leadership was measured using a 26-item scale (Li and Shi, 2008), capturing leaders’ behaviors related to meaning construction, vision articulation, intellectual stimulation, and individualized consideration. Consistent with prior research, the scale reflects four dimensions: moral modeling, articulating vision, individualized consideration, and charisma. Respondents evaluated the extent to which their immediate supervisors exhibited these behaviors within apprenticeship training and organizational activities. All items were rated on a 5-point Likert scale (1 = strongly disagree; 5 = strongly agree).

Organizational identification was measured using a 5-item scale assessing the degree to which respondents define themselves in terms of organizational membership and experience a sense of belonging and oneness (Smidts et al., 2001). The scale was translated into Chinese using a standard translation–back-translation procedure to ensure semantic equivalence. Items capture both affective attachment and cognitive alignment with organizational values and goals. All items were rated on a 5-point Likert scale (1 = strongly disagree; 5 = strongly agree).

In this study, the referent of “organization” was explicitly defined in the survey instructions as the enterprise unit in which apprentices conducted their work and training activities. Although apprentices remain formally affiliated with vocational colleges, their daily supervision, evaluation, and innovation-related activities occur within the enterprise context. The apprenticeship program operates as an integrated hybrid arrangement combining educational and enterprise logics within a single operational setting. Accordingly, identification with “this organization” captures alignment with the hybrid entity through which cross-boundary coordination is enacted.

Organizational innovation was assessed using a modified 11-item scale (Wang and Zhu, 2009), grounded in the conceptualization proposed by Damanpour and Evan (1984). Consistent with this framework, organizational innovation was operationalized as comprising managerial innovation and technological innovation. Items were rated on a 7-point Likert scale, with higher scores indicating greater levels of perceived innovation.

In this study, organizational innovation is conceptualized as perceived organizational innovation rather than objective archival output. This operationalization aligns with the study’s focus on leadership, identification, and identity-based coordination processes, which are inherently interpretive and experienced at the individual level. Apprentices are embedded in daily production, training, and administrative routines and are directly exposed to changes in work procedures, supervisory practices, coordination mechanisms, and technological initiatives. Accordingly, the innovation measures capture respondents’ observed and experienced innovation practices within their organizational context. Perceptual measures of organizational innovation are widely used in leadership and organizational research when examining interpretive and relational mechanisms.

To ensure construct clarity and measurement relevance in the context of apprenticeship-based hybrid organizations, we retained items that most directly reflected the core features of each innovation domain as experienced by the current sample. Managerial innovation was measured using six items capturing changes in management practices, administrative processes, and coordination routines. Technological innovation was measured using five items assessing the adoption, development, and application of new technologies, products, or production methods. These two dimensions are conceptually distinct, reflecting innovation in organizational processes vs. technical systems.

### 3.3 Mitigation of common method bias

Because the study relies on survey data from a single respondent group, multiple procedural and analytical steps were implemented to reduce the risk of common method bias (Podsakoff et al., 2024). Procedurally, participation was voluntary and anonymous, and respondents were assured that their answers would not be accessible to supervisors or used for evaluative purposes. Surveys were completed outside formal assessment contexts, reducing evaluation apprehension and social desirability pressures. In addition, the focal constructs were conceptually distinct: transformational leadership captured supervisory behaviors, organizational identification reflected self–organization alignment, and organizational innovation assessed perceived organizational practices rather than individual performance. Analytically, statistical modeling (see below) was employed to assess construct distinctiveness and to evaluate the potential influence of common method variance.

### 3.4 Analytical strategy

The hypotheses were tested using SEM, which enables the simultaneous estimation of latent constructs, direct and indirect relationships, and multiple outcome variables within a unified analytical framework. SEM is appropriate for the present study because transformational leadership, organizational identification, and organizational innovation are conceptualized as latent variables measured by multiple indicators, and because the theoretical model involves mediation and domain-specific differentiation of innovation outcomes.

All analyses were conducted in R using the lavaan package (version 0.6–21). Models were estimated using robust maximum likelihood (MLR) with full information maximum likelihood (FIML) to handle missing data. FIML utilizes all available information under the assumption of missing at random and is widely regarded as a rigorous approach in SEM applications.

Because respondents were nested within 45 institutions, we examined the extent of clustering effects prior to model estimation. Intraclass correlation coefficients (ICCs) were calculated using unconditional random-intercept models for each focal construct. ICC values were modest, indicating that the majority of variance resided at the individual level. Accordingly, single-level SEM was retained for subsequent analyses.

The analytical procedure proceeded in stages. First, confirmatory factor analysis (CFA) was conducted to evaluate the measurement model. All latent constructs were estimated simultaneously, including a second-order factor structure for transformational leadership. Model fit was evaluated using multiple complementary indices, including the standardized root mean square residual (SRMR), the comparative fit index (CFI), the Tucker–Lewis index (TLI), and the root mean square error of approximation (RMSEA), along with their robust counterparts appropriate for MLR estimation. Consistent with recent methodological recommendations (Mai et al., 2021), SRMR was treated as the primary indicator of absolute model fit, given its robustness in large-sample and complex SEM contexts.

Discriminant validity was assessed through nested model comparisons. Specifically, alternative measurement models were specified in which conceptually proximal constructs were collapsed into a single latent factor (e.g., merging managerial and technological innovation; merging transformational leadership and organizational identification). These nested models were compared with the baseline four-factor model using Satorra–Bentler scaled chi-square difference tests and changes in global fit indices. Significant deterioration in fit when constructs were merged was interpreted as evidence of empirical distinctiveness.

To further assess potential common method bias, a single-factor CFA model was estimated in which all measurement items loaded onto one latent factor. Substantially poorer fit of this model relative to the hypothesized four-factor structure was interpreted as evidence that the covariance structure is unlikely to be attributable to a single underlying method factor.

After establishing measurement adequacy, structural models were estimated. First, a baseline mediation model examined organizational identification as a mediator linking transformational leadership to overall organizational innovation. Direct and indirect paths were estimated simultaneously to evaluate partial mediation. Second, a differentiated structural model was specified in which managerial innovation and technological innovation were modeled as parallel outcome variables within the same SEM. Transformational leadership was specified to predict organizational identification, and both transformational leadership and organizational identification were specified to predict each innovation domain. Estimating both outcomes within a single model ensured that parameter estimates were directly comparable across domains while accounting for shared predictor variance.

Inference for mediation and cross-domain differences was conducted using nonparametric bootstrap resampling. Specifically, 5,000 bootstrap samples were drawn with replacement, and the full SEM was re-estimated for each resample. Standardized path coefficients were extracted at each iteration.

The standardized indirect effect of transformational leadership on innovation via organizational identification was computed as the product of the relevant path coefficients (Equation 1):


Indirecteffect=a×b, (1)

where a denotes the path from transformational leadership to organizational identification, and b denotes the path from organizational identification to the innovation outcome.

To assess domain differences, indirect effects for managerial and technological innovation were computed within each bootstrap sample, and their difference was calculated as (Equation 2):


$$\Delta_{\text{indirect}}=(a\times b_{\mathrm{MI}})-(a\times b_{\mathrm{TI}}),$$ (2)

where $b_{\mathrm{MI}}$ and $b_{\mathrm{TI}}$ represent the paths from organizational identification to managerial and technological innovation, respectively. Statistical inference for this difference relied on percentile bootstrap confidence intervals derived from the empirical distribution of standardized indirect differences across refitted bootstrap samples.

As a complementary analysis, differences in the direct effects of transformational leadership on managerial vs. technological innovation were also examined. The direct-effect difference was specified as (Equation 3):

Direct-effect differences between managerial and technological innovation were similarly evaluated within the SEM framework (Equation 3):


$$\Delta_{\text{direct}}=c_{\mathrm{MI}}-c_{\mathrm{TI}},$$ (3)

where $c_{\mathrm{MI}}$ and $c_{\mathrm{TI}}$ denote the direct paths from transformational leadership to managerial and technological innovation, respectively.

Primary structural models were estimated without demographic covariates, consistent with the theory-driven focus on relational and identity-based mechanisms at the individual perceptual level. The theoretical framework does not posit demographic heterogeneity as a boundary condition.

Nevertheless, to assess robustness, supplementary sensitivity analyses were conducted in which region and company type were included as exogenous covariates predicting organizational identification and both innovation outcomes. These analyses allowed us to evaluate whether the focal direct, indirect, and cross-domain effects were sensitive to organizational composition.

## 4 Results

### 4.1 Participants and survey statistics

Data were collected between March 1, 2025 and October 30, 2025. A total of 2,316 questionnaires were returned. After excluding 160 invalid responses—based on abnormally short response times, excessive missing values on key variables (exceeding 20%), or duplicate IP submissions—the final analytical sample consisted of 2,156 valid responses, yielding an effective response rate of 93.1%.

With respect to regional distribution, the majority of respondents were located in Hunan Province (83.12%), followed by Zhejiang (10.11%), Guangdong (2.92%), and other regions (3.85%). In terms of organizational affiliation, most respondents were drawn from automobile manufacturing enterprises (82.90%), while smaller proportions came from automobile technology firms (0.97%), new energy vehicle enterprises (2.04%), and automobile service-related organizations (14.01%). This composition mirrors the structure of China’s apprenticeship implementation in the automotive sector, which remains centered on manufacturing while increasingly incorporating technology-intensive and service-oriented segments. Overall, the regional and industry distributions of the sample are consistent with the current landscape of apprenticeship-based industry–education collaboration in China, supporting the representativeness and external validity of the data.

Table 1 reports the descriptive statistics and correlations for the main study variables. As shown in Table 1, transformational leadership was positively correlated with organizational identification, managerial innovation, and technological innovation. Organizational identification was also positively associated with both innovation domains. All correlations were statistically significant (*p* < 0.001).

**Table 1:** Descriptive statistics and correlations.

Variable	Mean	SD	1	2	3	4
1. Transformational Leadership	4.10	0.85	—			
2. Organizational Identification	4.07	0.85	0.88	—		
3. Managerial Innovation	5.70	1.28	0.89	0.88	—	
4. Technological Innovation	5.76	1.28	0.88	0.91	0.94	—

*N =* 2,156. All correlations are significant at *p* < 0.001.

Given that participants were nested within 45 institutions, we examined the degree of between-institution variance for the primary constructs. Intraclass correlation coefficients were modest (transformational leadership = 0.044; organizational identification = 0.041; managerial innovation = 0.027; technological innovation = 0.038), indicating that only a small proportion of variance was attributable to institutional clustering. These values suggest that the constructs primarily reflect individual-level perceptions, supporting the use of single-level SEM in subsequent analyses.

### 4.2 Measurement model

A CFA was conducted to evaluate the adequacy of the measurement model prior to testing the hypothesized structural relationships. The model specified four latent constructs—transformational leadership (modeled as a second-order factor reflected by four first-order dimensions), organizational identification, managerial innovation, and technological innovation—each represented by their respective indicators.

The baseline four-factor measurement model demonstrated satisfactory fit to the data. As reported in Table 2, the SRMR was well below the 0.06 threshold. In addition, both the robust CFI and robust TLI exceeded conventional benchmarks for acceptable fit, and the robust RMSEA fell within the range typically regarded as reasonable for complex latent variable models. Considered jointly, the pattern of robust fit indices supports the adequacy of the proposed measurement structure.

**Table 2:** Measurement model fit and nested model comparisons.

Model	Robust CFI	Robust TLI	Robust RMSEA	SRMR	χ^2^	Δχ^2^
Baseline four-factor model	0.931	0.927	0.085	0.018	4647.8	—
MI–TI merged model	0.925	0.921	0.088	0.019	4865.3	391.03***
TL–OID merged model	0.908	0.902	0.098	0.028	5470.0	96.20***
Single-factor model	0.820	0.811	0.136	0.039	8481.1	258.32***

Robust fit indices are based on maximum likelihood estimation with robust standard errors. χ^2^ and Δχ^2^ values reflect Satorra–Bentler scaled chi-square statistics. CFI, comparative fit index; TLI, Tucker–Lewis index; RMSEA, root mean square error of approximation; SRMR, standardized root mean square residual. TL, transformational leadership; OID, organizational identification; MI, managerial innovation; TI, technological innovation. ***p < 0.001.

To assess discriminant validity, alternative nested measurement models were specified and compared with the baseline four-factor model. As shown in Table 2, collapsing managerial and technological innovation into a single factor resulted in a statistically significant deterioration in model fit. Likewise, combining transformational leadership and organizational identification led to a substantial worsening of fit. These findings indicate that the four constructs are empirically separable at the latent level.

To further evaluate whether the covariance structure could be attributed to a single underlying factor, a single-factor CFA model was estimated in which all measurement items loaded onto one latent construct. This model exhibited substantially poorer fit relative to the baseline four-factor model, and the Satorra–Bentler scaled chi-square difference test was significant. The pronounced degradation in fit suggests that the observed covariance among indicators cannot be adequately explained by a single general factor.

Although the zero-order correlations reported in Table 1 are high, discriminant validity in SEM is evaluated at the latent level rather than on the basis of bivariate correlations alone. In apprenticeship-based hybrid organizations characterized by tightly integrated supervision, collective identity formation, and coordinated innovation initiatives, strong covariation among transformational leadership, identification, and innovation perceptions is theoretically plausible. Importantly, however, the superior fit of the four-factor model relative to both conceptually collapsed models and the single-factor specification demonstrates that these constructs are not empirically redundant.

Internal consistency reliability (Table 3) was assessed using Cronbach’s alpha and McDonald’s omega coefficients. All constructs demonstrated strong reliability, with coefficients exceeding recommended thresholds. Convergent validity (Table 3) was evaluated using the average variance extracted (AVE), and all AVE values exceeded 0.50. In addition, for each construct, the AVE exceeded the squared correlations with other latent variables, further supporting discriminant validity.

**Table 3:** Reliability results of the four-factor model.

Construct	McDonald’s Omega	Cronbach’s α	AVE
Transformational Leadership	—	0.994	0.821
Organizational Identification	0.968	0.967	0.855
Managerial Innovation	0.977	0.977	0.878
Technological Innovation	0.976	0.977	0.893

Taken together, the CFA results, nested model comparisons, single-factor test, and reliability and validity diagnostics provide converging evidence that transformational leadership, organizational identification, managerial innovation, and technological innovation represent empirically distinct constructs within the present sample. The four-factor measurement structure was therefore retained for subsequent structural analyses.

### 4.3 Baseline structural model: organizational identification as a mediator

Having established the adequacy of the measurement model, we next estimated the baseline structural model to examine the hypothesized mediation framework linking transformational leadership, organizational identification, and organizational innovation. This model evaluates the proposed relational mechanism at the aggregate innovation level prior to differentiating between innovation domains.

As shown in Table 4, transformational leadership was positively and strongly associated with organizational identification. Organizational identification, in turn, was positively associated with organizational innovation. Transformational leadership also retained a significant direct association with organizational innovation, indicating that leadership is related to innovation both directly and indirectly through identification processes.

**Table 4:** Baseline structural model results.

Effect type	Path	*β* [95% CI]
Direct	Transformational Leadership → Organizational Identification	0.898 [0.853, 0.930] ***
Direct	Organizational Identification → Organizational Innovation	0.594 [0.410, 0.772] ***
Direct	Transformational Leadership → Organizational Innovation	0.380 [0.194, 0.568] ***
Indirect	Transformational Leadership → Organizational Identification → Organizational Innovation	0.533 [0.364, 0.702] ***
Total	Transformational Leadership → Organizational Innovation	0.913 [0.876, 0.941] ***

Standardized path coefficients (β) are reported with associated confidence intervals (CIs). ***p < 0.001.

Consistent with the hypothesized mediation logic, the indirect effect of transformational leadership on organizational innovation via organizational identification was positive and statistically significant (*β* = 0.533, *p* < 0.001). The persistence of a significant direct path alongside a significant indirect effect is consistent with partial mediation.

The total effect of transformational leadership on organizational innovation was statistically significant. Taken together, these results are consistent with the proposed mediation mechanism and provide a baseline structure for subsequent domain-specific analyses.

### 4.4 Differentiated structural model: managerial vs. technological innovation

Building on the baseline mediation model, we next estimated a differentiated structural model in which organizational innovation was disaggregated into managerial and technological domains. Both outcomes were modeled simultaneously within a single SEM framework, allowing domain-specific mediation patterns to be evaluated while holding the broader relational structure constant.

The differentiated structural model demonstrated satisfactory fit to the data (SRMR < 0.02). As shown in Table 5, transformational leadership remained strongly and positively associated with organizational identification. Organizational identification was positively associated with both managerial and technological innovation. Transformational leadership also exhibited statistically significant direct associations with each innovation domain, indicating that leadership is related to both forms of innovation beyond identification-based pathways.

**Table 5:** Differentiated structural model results: managerial vs. technological innovation.

Effect type	Path	*β* [95% CI]
Direct	Transformational Leadership → Organizational Identification	0.897 [0.853, 0.930] ***
Direct	Organizational Identification → Managerial Innovation	0.443 [0.235, 0.667] ***
Direct	Transformational Leadership → Managerial Innovation	0.508 [0.284, 0.714] ***
Direct	Organizational Identification → Technological Innovation	0.667 [0.495, 0.839] ***
Direct	Transformational Leadership → Technological Innovation	0.298 [0.115, 0.473] **
Indirect	Transformational Leadership → Organizational Identification → Managerial Innovation	0.398 [0.207, 0.605] ***
Indirect	Transformational Leadership → Organizational Identification → Technological Innovation	0.598 [0.440, 0.759] ***
Total	Transformational Leadership → Managerial Innovation	0.906 [0.870, 0.933] ***
Total	Transformational Leadership → Technological Innovation	0.897 [0.855, 0.928] ***

Standardized path coefficients (β) are reported with associated confidence intervals (CIs). **p < 0.01; ***p < 0.001.

Mediation analyses revealed significant indirect associations of transformational leadership with managerial innovation (*β* = 0.398, *p* < 0.001) and technological innovation (*β* = 0.598, *p* < 0.001) through organizational identification, consistent with partial mediation. Notably, the magnitude of the indirect pathway differed across domains.

To formally evaluate whether the mediation pattern varied across innovation domains, we compared the two indirect effects within the SEM framework. As reported in Table 6, the difference between the indirect effects was statistically significant, indicating that the identification-based pathway is stronger for technological innovation than for managerial innovation.

**Table 6:** Difference tests for indirect and direct effects across innovation domains.

Effect type	Difference tested	Estimate [95% CI]	*p*-value
Indirect	(TL → OID → MI) − (TL → OID → TI)	−0.201 [−0.353, −0.041]	0.011
Direct	(TL → MI) − (TL → TI)	0.210 [0.034, 0.383]	0.017

Standardized estimates are reported with associated confidence intervals (CIs). Negative values indicate larger effects for technological innovation relative to managerial innovation. TL, transformational leadership; OID, organizational identification; MI, managerial innovation; TI, technological innovation.

We also examined cross-domain differences in the direct associations of transformational leadership. The direct association with managerial innovation exceeded that with technological innovation, and the difference between these paths was statistically significant (Table 6), suggesting domain-specific variation in the non-mediated pathway linking leadership and innovation.

Taken together, the differentiated model reveals both shared and domain-specific patterns. Transformational leadership is associated with managerial and technological innovation through partially overlapping pathways, with identification-based processes showing a comparatively stronger association with technological innovation, while direct leadership associations are more pronounced for managerial innovation.

### 4.5 Robustness check

To assess whether the observed structural relationships were sensitive to organizational composition, supplementary models were estimated including region and company type as exogenous covariates.

For the baseline mediation model, inclusion of these covariates did not materially alter the magnitude, direction, or statistical significance of the focal direct, indirect, or total associations (see Supplementary Table S1). Standardized path coefficients remained substantively similar, and the mediation pattern was preserved.

For the differentiated structural model, the domain-specific pattern likewise remained stable (see Supplementary Table S2). The indirect association of transformational leadership via organizational identification continued to be stronger for technological innovation than for managerial innovation, while the direct association remained stronger for managerial innovation. Changes in standardized coefficients were minimal, and focal effects retained statistical significance.

Collectively, these findings indicate that the mediation mechanism and the domain-differentiated pattern are robust to variation in regional distribution and company-type composition within the sample.

## 5 Discussion

This study examined patterns of coordinated innovation in apprenticeship-based hybrid organizations characterized by institutional plurality, fragmented authority, and overlapping role expectations.

Moving beyond whether transformational leadership is associated with innovation, the analysis focused on how leadership is linked to innovation through organizational identification. Drawing on social identity theory, the study examines whether leadership influences innovation by fostering shared organizational identification among members operating within hybrid organizational contexts characterized by institutional plurality. The findings indicate that organizational identification is positively associated with the relationship between transformational leadership and innovation outcomes, and that the relative strength of mediated and direct associations differs between managerial and technological innovation.

### 5.1 Transformational leadership, organizational identification, and innovation in hybrid organizations

The mediation findings suggest that organizational identification plays an important role in linking transformational leadership with innovation in apprenticeship-based hybrid organizations. This pattern is consistent with prior research indicating that hybrid organizations face coordination challenges that are fundamentally social and interpretive, as members navigate competing institutional logics and ambiguous role expectations in the absence of unified authority structures (Battilana and Lee, 2014; Besharov and Smith, 2014). In this context, leadership appears to be associated less with direct control and more with processes that foster shared understanding and collective orientation.

Previous studies have documented positive associations between transformational leadership and innovation (Jung et al., 2003; Gumusluoglu and Ilsev, 2009). The present findings extend this literature by clarifying how such associations manifest in apprenticeship-based hybrid organizations. Specifically, the results suggest that the association between leadership and innovation is partially transmitted through organizational identification, indicating that identification may function as an important pathway through which leadership is linked to innovation when authority relations and organizational boundaries are fragmented.

At the same time, the persistence of a significant direct association between transformational leadership and organizational innovation indicates that leadership–innovation relationships are not fully accounted for by identification-related processes. Beyond fostering shared identification, leaders may contribute to innovation by legitimizing experimentation, framing change as appropriate, and directing attention toward innovation-related activities (Podsakoff et al., 1990; Waldman et al., 2001). The coexistence of mediated and direct pathways suggests that leadership–innovation associations likely operate through multiple complementary mechanisms in hybrid organizational environments.

From a social identity theory perspective, these findings suggest that leadership influence in hybrid organizations operates partly through the construction of shared organizational identity. Social identity theory proposes that individuals are more likely to coordinate their behavior and support collective goals when they perceive themselves as members of a common social group (Ashforth and Mael, 1989). In apprenticeship-based hybrid organizations, where members simultaneously navigate educational and industrial roles, leadership behaviors that reinforce organizational identification may help reduce identity fragmentation and align members’ actions around innovation-related goals. The results therefore provide empirical support for the argument that identity-based processes play an important role in enabling coordinated innovation under conditions of institutional plurality.

### 5.2 Differentiating managerial and technological innovation

Disaggregating organizational innovation into managerial and technological domains provides additional insight into how leadership and identification are associated with innovation outcomes in hybrid organizations. From a social identity theory perspective, different forms of innovation may place distinct demands on identity-based coordination. Innovations that depend heavily on collaborative problem solving and cross-boundary knowledge integration may rely more strongly on shared identification among organizational members, whereas innovations involving administrative structures may allow greater scope for direct leadership intervention.

Organizational identification mediates the relationship between transformational leadership and both managerial and technological innovation, but the mediated association is stronger for technological innovation. This pattern suggests that technological innovation—characterized by uncertainty, experimentation, and cross-boundary knowledge integration—is particularly sensitive to identity-based coordination. In apprenticeship-based hybrid organizations, apprentices are directly embedded in production processes and technical workflows, where innovation often depends on discretionary engagement, collaborative problem solving, and the integration of learning with practice. Because technological innovation unfolds within day-to-day operational contexts, shared identification may be especially critical for sustaining engagement, reducing identity-related friction, and encouraging cross-boundary knowledge exchange (Gioia et al., 2013; Hetemi et al., 2026; Ungureanu et al., 2020). In this sense, apprentices’ proximity to technical tasks may amplify the importance of identification as a mechanism through which leadership influence translates into technological innovation.

In contrast, the direct association between transformational leadership and innovation is stronger for managerial innovation than for technological innovation. Managerial innovation typically involves changes in administrative routines, management practices, and coordination mechanisms, which may allow leaders to exert more immediate influence through formal decision making, role modeling, and the explicit legitimation of new practices. Administrative changes are often initiated and structured by leadership authority, making them more directly responsive to leader-driven advocacy and framing. This pattern is consistent with arguments that administrative innovation is more amenable to top-down intervention, whereas technological innovation depends more heavily on sustained collective engagement and shared interpretive alignment among organizational members (Volberda et al., 2013; Maitlis and Christianson, 2014; Bunjak et al., 2022; Rohlfer et al., 2022).

Taken together, these findings refine the mechanism–task alignment perspective advanced in this study. When innovation demands are closely tied to operational tasks and cross-boundary knowledge integration, identity-based coordination appears to be especially consequential. When innovation primarily concerns administrative structures and formal routines, leadership shows a comparatively stronger direct association. This differentiation highlights that the relative importance of mediated vs. direct leadership pathways may vary depending on the coordination demands inherent in specific innovation domains.

### 5.3 Apprenticeship-based hybridity and domain-contingent coordination

The differentiated findings are particularly informative in the context of apprenticeship-based hybrid organizations. These arrangements integrate educational and industrial logics within a single organizational setting, distributing authority across firms, educational institutions, mentors, and supervisors. Participants simultaneously occupy roles as learners and workers, exposing them to overlapping and sometimes competing expectations regarding performance, learning, and organizational membership. Prior research suggests that such identity complexity heightens the importance of processes that help individuals achieve coherence across roles (Awasthy and Adya, 2025).

Within this setting, the findings indicate that organizational identification is closely associated with how leadership influence is translated into innovation outcomes. Identification-related processes appear especially salient for technological innovation, where coordination demands are high and collaboration across institutional boundaries is essential. At the same time, leadership influence is more directly associated with managerial innovation, which is more closely tied to organizational routines and administrative structures. Taken together, these patterns illustrate how leadership and identification interact empirically with the demands of different innovation domains in apprenticeship-based hybrid organizations.

## 6 Theoretical contributions

This study advances research on hybrid organizations by developing an identity-based explanation of how coordinated innovation becomes possible under conditions of institutional plurality. Existing scholarship on hybrid organizing has largely approached coordination challenges from a structural and institutional perspective, emphasizing governance arrangements, selective coupling, and organizational design as primary mechanisms for stabilizing hybridity (Battilana and Dorado, 2010; Pache and Santos, 2013; Smith and Besharov, 2019). While these work have generated important insights into how competing institutional logics can coexist, they have paid comparatively limited attention to the micro-level social processes through which organizational members align their actions and sustain collective innovation when authority structures and evaluative criteria are fragmented. By foregrounding leadership and organizational identification, this study shifts the theoretical focus from structural solutions to identity-based coordination as a central mechanism of innovation in hybrid organizations.

A first contribution lies in reconceptualizing transformational leadership in hybrid contexts and explicitly revising its boundary conditions. Prior leadership research has consistently associated transformational leadership with innovation by emphasizing its motivational, cognitive, and relational effects on followers (Qu et al., 2015; Sheehan et al., 2020). However, this literature has largely been developed in organizational contexts characterized by relatively centralized authority and coherent organizational identities. Within such settings, transformational leadership operates primarily by amplifying motivation, strengthening commitment, and enhancing alignment around institutionally stable goals. Hybrid organizations systematically violate these assumptions. Authority is distributed across organizations and professions, role expectations are ambiguous, and actors are embedded simultaneously in multiple institutional domains. Under such conditions, alignment cannot be presumed; it must be actively constructed. Leadership influence therefore cannot be fully understood as a function of directive power or incentive alignment alone.

This study provides evidence consistent with the argument that in pluralistic institutional environments, transformational leadership is closely associated with processes of sensemaking and identity integration. Leaders appear to construct shared interpretations of purpose and legitimacy, reconcile competing institutional logics, and stabilize expectations across fragmented roles. By theorizing and empirically examining this mechanism, the study extends leadership theory beyond its traditional motivational framing and positions identity integration—not merely control—as a key pathway linking leadership and collective action in complex organizational settings.

A second contribution concerns the theoretical role of organizational identification in hybrid organizing. Organizational identification has traditionally been conceptualized as an individual-level psychological attachment that enhances commitment and discretionary effort (Ashforth and Mael, 1989). While this view has been productive in relatively bounded organizations, it is insufficient for explaining coordination in hybrid contexts, where individuals must navigate overlapping and sometimes conflicting institutional identities (Kreiner et al., 2006).

This study advances a reconceptualization of organizational identification as a coordination infrastructure—a shared interpretive and normative framework that enables actors to align expectations, legitimize collaboration, and commit to collective courses of action despite institutional plurality. From this perspective, identification is not merely an attitudinal outcome but a foundational social condition that reduces uncertainty and stabilizes cooperation in the absence of unified authority. By linking social identity theory (Ashforth and Mael, 1989; Kogut and Zander, 1996) with hybrid organization research, the study provides a micro-level explanation of how institutional plurality becomes workable in everyday organizational practice. This micro-coordination perspective complements existing structural accounts and advances hybrid organization theory by illuminating the identity processes that sustain coordinated innovation. An important implication of this reconceptualization concerns the referent of identification in hybrid contexts. In apprenticeship-based hybrid organizations, the focal enterprise unit operates as the primary locus where educational and industrial logics are practically integrated. Apprentices’ daily supervision, evaluation, and innovation-related activities occur within this enterprise-based hybrid arrangement. Accordingly, identification with “the organization” in this context reflects identification with a structurally integrated hybrid entity rather than attachment to a single institutional domain. While individuals may retain formal ties to educational institutions, the operational site of coordination is the enterprise-based apprenticeship system itself. Thus, organizational identification in this study captures alignment with the cross-boundary hybrid arrangement through which coordinated innovation is enacted. Future research may extend this work by simultaneously measuring identification with multiple institutional referents (e.g., enterprise, educational institution, and apprenticeship program) to more directly assess identity plurality and integration dynamics.

A third contribution lies in advancing a domain-contingent view of innovation in hybrid organizations. Although organizational innovation has long been recognized as multidimensional, encompassing both managerial and technological forms (Damanpour and Evan, 1984), prior research often assumes that leadership and coordination mechanisms operate uniformly across innovation domains. This study challenges that assumption by theorizing that different forms of innovation place distinct demands on coordination mechanisms in hybrid settings.

Technological innovation, characterized by high uncertainty, experimentation, and cross-boundary knowledge integration, relies more heavily on identity-based coordination to legitimize risk taking and sustain collaboration across institutional divides. Managerial innovation, by contrast, is more closely tied to administrative routines and organizational authority, allowing greater scope for direct leadership intervention. By articulating and empirically examining these differences, the study contributes a mechanism–task alignment perspective to innovation theory, demonstrating that the effectiveness of leadership and identity-based processes depends on the coordination demands inherent in specific innovation domains.

Collectively, these contributions suggest that coordinated innovation under institutional plurality may not be fully explained by structural design or governance arrangements alone. Rather, identity-based processes appear to play a significant role in shaping how leadership is linked to innovation outcomes across domains.

## 7 Managerial implications

The findings of this study offer several implications for leaders and designers of apprenticeship-based hybrid organizations and, more broadly, for organizations operating across institutional boundaries. Hybrid arrangements promise innovation by combining diverse resources and expertise, yet they also intensify coordination challenges because authority, evaluation standards, and professional identities are distributed across multiple institutional domains. The results suggest that managerial effectiveness in such contexts may depends less on formal control and more on leaders’ capacity to align meaning and identity across institutional boundaries.

Leadership in hybrid organizations may therefore be productively understood as an ongoing process of identity integration rather than solely as an administrative or motivational function. Transformational leadership behaviors—such as articulating shared purpose, modeling collective values, and linking individual roles to overarching organizational goals—appear to be closely associated with coordinated action when formal authority is fragmented. For managers, this suggests that investing in leadership development focused on sensemaking, communication, and identity work may be particularly valuable, especially for supervisors and mentors operating at the intersection of educational and organizational systems. At the same time, identity-oriented leadership requires sustained interaction and organizational support, which may vary across hybrid settings.

The findings further indicate that innovation initiatives in hybrid organizations may benefit from sensitivity to the nature of the innovation task itself. Technological innovation, which often involves uncertainty, experimentation, and cross-boundary knowledge integration, appears to be more strongly associated with shared organizational identification. Managers seeking to promote technological upgrading or experimentation may therefore benefit from cultivating shared identity and collective ownership through practices such as cross-boundary project teams, joint training programs, and consistent symbolic messaging across institutional partners. Without such identity alignment, technological initiatives may encounter coordination difficulties even when technical resources are sufficient.

Managerial innovation, by contrast, is more closely tied to administrative routines and coordination structures, and the findings suggest that leadership exhibits a comparatively stronger direct association with this domain. Leaders may therefore be positioned to play a more direct role in initiating and implementing changes to management processes, performance systems, and organizational routines, even within hybrid settings. Recognizing these domain differences may help managers allocate attention and leadership effort more strategically rather than assuming that all forms of innovation require identical leadership approaches.

Overall, these implications highlight the importance of moving beyond one-size-fits-all approaches to leadership and innovation in hybrid organizations. Apprenticeship-based systems, in particular, may benefit from tailoring leadership practices to the specific coordination demands associated with different innovation goals. Viewing organizational identification as a strategic organizational resource—rather than merely a byproduct of organizational life—may assist managers in leveraging the innovative potential of hybrid arrangements while navigating the coordination challenges associated with institutional plurality.

## 8 Strength and limitations

Two features of the research design constitute key strengths of this study. First, China’s apprenticeship-based hybrid organizations provide a rigorous setting for examining leadership and organizational identification under institutional plurality. Apprentices’ dual embeddedness in enterprise and educational contexts exposes them to fragmented authority and overlapping role expectations, offering strong analytical leverage for studying identity-based coordination in pluralistic environments. Second, the study was conducted within Geely, a large and innovation-intensive enterprise. Examining these relationships in a context characterized by active technological upgrading increases confidence that the observed associations among leadership, identification, and innovation perceptions are situated in a setting where innovation is organizationally consequential.

At the same time, several limitations warrant careful consideration. First, the study is situated within the institutional context of China’s apprenticeship-based hybrid system, which is shaped by strong state involvement and distinctive governance arrangements. Although this setting offers theoretical leverage for examining coordination under institutional plurality, future research should examine whether the identity-based mechanisms identified here generalize to hybrid organizations operating in different national or institutional environments. Comparative studies across countries or sectors would help clarify the boundary conditions of identity-integrating leadership.

Second, the cross-sectional design precludes firm causal inference. Although the theoretical model specifies directional relationships, the observed associations cannot establish temporal precedence or rule out reciprocal effects among leadership, identification, and innovation perceptions. Longitudinal or experimental research would be necessary to more rigorously assess causal dynamics.

Third, the study relies on self-reported perceptions from apprentices. While appropriate for capturing leadership behaviors and identity processes, single-source data raise concerns about common method variance. Although procedural safeguards and measurement-model tests were implemented, future research should incorporate multi-source designs, archival indicators, or temporally separated measures.

Fourth, zero-order correlations among focal constructs are high. Respondents who evaluate leadership positively may also hold generally favorable views of organizational functioning, contributing to common evaluative tendencies. Although CFA supports latent discriminant validity, strong covariation may inflate effect magnitudes and warrants cautious interpretation. Multi-wave designs or alternative operationalizations would help further disentangle these constructs.

Fifth, respondents were nested within 45 institutions. Although intraclass correlations were modest, unmodeled contextual influences may remain. Multilevel designs would allow future research to examine cross-level dynamics more explicitly.

Sixth, the study focuses on apprentices and does not capture mentors’ perspectives—actors who carry formal authority and boundary-spanning responsibilities. Incorporating mentors would enable a more role-differentiated understanding of identity-integrating leadership in hybrid settings.

Finally, organizational identification was measured with respect to the focal enterprise rather than multiple institutional referents. Although the apprenticeship system functions as an integrated hybrid arrangement, future research could simultaneously assess identification with enterprise, educational institutions, and the apprenticeship program to more directly examine identity plurality and integration dynamics.

## 9 Conclusion

Hybrid organizations promise innovation by combining diverse resources and institutional logics, yet they also face persistent coordination challenges arising from fragmented authority and plural identities. This study provides evidence consistent with the view that coordinated innovation in such contexts is closely associated not only with formal structures or governance arrangements, but also with leadership-linked identity integration. By differentiating managerial and technological innovation, the findings further suggest that leadership–innovation relationships vary across domains, underscoring the importance of aligning coordination mechanisms with the specific demands of different innovation tasks. As hybrid organizational forms continue to proliferate across sectors, understanding how leadership and identification jointly shape innovation processes remains a central theoretical and practical challenge.

## Data Availability

The raw data supporting the conclusions of this article will be made available by the authors, without undue reservation.
