# Supplementary material for: Coordinating innovation without unified authority: leadership and organizational identification in hybrid organizations
Source: Front Psychol. 2026 Apr 2;17:1803166. doi: 10.3389/fpsyg.2026.1803166 (PMC13082938; doi:10.3389/fpsyg.2026.1803166)
Supplement: Supplementary file 1 [file Table_1.DOCX]

**Supplementary Box 1. List of Survey Questionnaires.**

| **Transformational Leadership (TL)** |
| --- |
| TL:moral modeling Q1: My supervisor or mentor acts with integrity and does not seek personal gain. |
| TL:moral modeling Q2: My supervisor or mentor is willing to endure hardship before others and enjoy benefits afterward. |
| TL:moral modeling Q3: My supervisor or mentor puts aside personal concerns and works wholeheartedly. |
| TL:moral modeling Q4: My supervisor or mentor is willing to sacrifice personal interests for the benefit of the department or organization. |
| TL:moral modeling Q5: My supervisor or mentor places collective and others’ interests above personal interests. |
| TL:moral modeling Q6: My supervisor or mentor does not appropriate others’ work achievements as their own. |
| TL:moral modeling Q7: My supervisor or mentor shares difficulties and successes with employees. |
| TL:moral modeling Q8: My supervisor or mentor does not unfairly target or retaliate against employees. |
| TL:articulate vision Q1: My supervisor or mentor helps employees understand the future prospects of the department or organization. |
| TL:articulate vision Q2: My supervisor or mentor helps employees understand the organization’s philosophy and development goals. |
| TL:articulate vision Q3: My supervisor or mentor explains the long-term significance of the work we do. |
| TL:articulate vision Q4: My supervisor or mentor presents an inspiring vision of the future. |
| TL:articulate vision Q5: My supervisor or mentor provides clear goals and direction for employees to strive toward. |
| TL:articulate vision Q6: My supervisor or mentor frequently discusses how employees’ work contributes to the overall goals of the department or organization. |
| TL:individualized consideration Q1: My supervisor or mentor takes employees’ personal circumstances into account when interacting with them. |
| TL:individualized consideration Q2: My supervisor or mentor is willing to help employees address difficulties in their personal or family life. |
| TL:individualized consideration Q3: My supervisor or mentor communicates frequently with employees to understand their work, life, and family situations. |
| TL:individualized consideration Q4: My supervisor or mentor patiently guides employees and answers their questions. |
| TL:individualized consideration Q5: My supervisor or mentor cares about employees’ work, life, and development, and sincerely offers advice for their growth. |
| TL:individualized consideration Q6: My supervisor or mentor creates opportunities for employees to fully utilize their strengths. |
| TL:charisma Q1: My supervisor or mentor demonstrates strong professional competence. |
| TL:charisma Q2: My supervisor or mentor is open-minded and has a strong sense of innovation. |
| TL:charisma Q3: My supervisor or mentor is passionate about their work and highly committed to their career. |
| TL:charisma Q4: My supervisor or mentor is deeply engaged in their work and consistently maintains a high level of enthusiasm. |
| TL:charisma Q5: My supervisor or mentor continuously learns in order to improve themselves. |
| TL:charisma Q6: My supervisor or mentor is decisive and capable of handling difficult problems. |
| **Organizational Identification (OID)** |
| OID:Q1: I feel that my fate is closely tied to that of my organization. |
| OID:Q2: I feel a strong sense of belonging to my organization. |
| OID:Q3: I feel proud to work for this organization. |
| OID:Q4: I strongly identify with this organization. |
| OID:Q5: I feel honored to be a member of this organization. |
| **Organizational Innovation (OI)** |
| OI:managerial innovation Q1: My organization actively adopts new measures to improve organizational performance. |
| OI:managerial innovation Q2: My organization improves work processes or methods to enhance efficiency. |
| OI:managerial innovation Q3: My organization adopts new compensation systems to better motivate employees. |
| OI:managerial innovation Q4: My organization demonstrates distinctive insights regarding its future development. |
| OI:managerial innovation Q5: My organization formulates contingency plans in a timely manner in response to environmental changes. |
| OI:managerial innovation Q6: My organization adjusts departmental structures and employee performance management in response to practical conditions. |
| OI:technological innovation Q1: My organization updates the content and forms of its services in response to customer needs. |
| OI:technological innovation Q2: My organization introduces new technologies and equipment to improve productivity. |
| OI:technological innovation Q3: My organization adopts new technologies to optimize work processes. |
| OI:technological innovation Q4: My organization develops new products that meet market demands. |
| OI:technological innovation Q5: My organization updates and integrates its knowledge resources in a timely manner. |

**Supplementary Table S1. Baseline Structural Model with Demographic Controls.**

| **Effect Type** | **Path** | **Baseline *β*** | **Controlled *β*** | **Δ*β* (Controlled − Baseline)** |
| --- | --- | --- | --- | --- |
| Direct | TL → OID | 0.898 | 0.898 | 0 |
| Direct | OID → OI | 0.594 | 0.599 | 0.005 |
| Direct | TL → OI | 0.380 | 0.375 | -0.005 |
| Indirect | TL → OID → OI | 0.533 | 0.538 | 0.005 |
| Total | TL → OI | 0.913 | 0.913 | 0 |

Note. *β* = standardized path coefficient. Δ*β* = difference between controlled and baseline estimates. TL = Transformational Leadership; OID = Organizational Identification; OI = Organizational Innovation. Controlled model includes Region and Company type as covariates predicting OID and OI. All focal effects remain statistically significant at *p* < 0.001 in both specifications.

**Supplementary Table S2. Differentiated Structural Model with Demographic Controls**

| **Effect Type** | **Path** | **Baseline *β*** | **Controlled *β*** | **Δ*β* (Controlled − Baseline)** |
| --- | --- | --- | --- | --- |
| Direct | TL → OID | 0.897 | 0.898 | 0.001 |
| Direct | OID → MI | 0.443 | 0.454 | 0.011 |
| Direct | OID → TI | 0.667 | 0.669 | 0.002 |
| Direct | TL → MI | 0.508 | 0.498 | -0.01 |
| Direct | TL → TI | 0.298 | 0.296 | -0.002 |
| Indirect | TL → OID → MI | 0.398 | 0.407 | 0.009 |
| Indirect | TL → OID → TI | 0.598 | 0.601 | 0.003 |
| Total | TL → MI | 0.906 | 0.905 | -0.001 |

Note. *β* = standardized path coefficient. Δ*β* = difference between controlled and baseline estimates. TL = Transformational Leadership; OID = Organizational Identification; OI = Organizational Innovation. Controlled model includes Region and Company type as covariates predicting OID and OI. All focal effects remain statistically significant at *p* < 0.01 in both specifications.
